# Marburg Virus Infection in Egyptian Rousette Bats, South Africa, 2013–2014^1^

**DOI:** 10.3201/eid2406.172165

**Published:** 2018-06

**Authors:** Janusz T. Pawęska, Petrus Jansen van Vuren, Alan Kemp, Nadia Storm, Antoinette A. Grobbelaar, Michael R. Wiley, Gustavo Palacios, Wanda Markotter

**Affiliations:** National Institute for Communicable Diseases of the National Health Laboratory Service, Johannesburg, South Africa (J.T. Pawęska, P. Jansen van Vuren, A. Kemp, N. Storm, A.A. Grobbelaar);; University of Pretoria, Pretoria, South Africa (J.T. Pawęska, P. Jansen van Vuren, N. Storm, W. Markotter);; University of Nebraska Medical Center, Omaha, Nebraska, USA (M.R. Wiley);; US Army Medical Research Institute of Infectious Diseases, Frederick, Maryland, USA (M.R. Wiley, G. Palacios)

**Keywords:** Marburg virus, fruit bats, South Africa, Egyptian rousette bats, seroprevalence, viruses, Matlapitsi Cave, mating season, birthing season, Ozolin strain

## Abstract

We detected a high seroprevalence of Marburg virus (MARV) antibodies in fruit bats in South Africa; 19.1% of recaptured bats seroconverted. The MARV RNA isolated closely resembled the 1975 Ozolin strain. These findings indicate endemic MARV circulation in bats in South Africa and should inform policies on MARV disease risk reduction.

As of March 2018, thirteen outbreaks of Marburg virus (MARV) disease (MVD) have been reported, most occurring in sub-Saharan Africa (*1*,*2*). The first recognized outbreak of MVD in Africa occurred in 1975 after a person hitchhiking through Zimbabwe was admitted to Johannesburg Hospital, Johannesburg, South Africa (*3*). The largest MVD outbreak occurred in Angola during 2004–2005 and had a case-fatality rate of 90% (*4*).

Outbreaks of MVD in Africa have been associated with persons entering caves or mines (*5*–*8*), and results of outbreak investigations and ecologic and experimental studies implicate the Egyptian rousette bat (*Rousettus aegyptiacus*) as the prime reservoir host for MARV (*9*–*13*). As part of a biosurveillance program in South Africa investigating the presence of viral zoonotic pathogens in bats, we tested a local population of Egyptian rousette bats for evidence of MARV infection.

## The Study

At monthly intervals during February 2013–February 2014, we captured and sampled Egyptian rousette bats at the entrance to Matlapitsi Cave, also known as Mahune Cave. The cave is located in the indigenous flora of Matlapitsi Valley (24°1′S, 30°10′E) on the northeastern slope of the Wolkberg mountain range, bordering Lekgalameetse Nature Reserve in Limpopo Province, South Africa. This work was done in accordance with approved protocols by animal ethics committees of the National Health Laboratory Service (Johannesburg, South Africa; AEC 137/12) and the University of Pretoria (Pretoria, South Africa; EC054–14). We captured and handled bats using standard procedures (*10*) and determined sex and age by visual evaluation of size, pelage color, and reproductive status. We tattooed and sampled anesthetized bats and processed blood and tissue specimens as described previously (*12*). We collected blood samples monthly from a subset (n = 1,431) of the total population of Egyptian rousette bats sampled during the 13-month biosurveillance program. Blood sample collection varied from 61 samples/month (April 2013) to 197 samples/month (May 2013). In addition, we collected spleen and liver tissues from 159 bats (average collection rate 12 samples/month). During the study period, 63 bats were recaptured (average rate 5 bats/month). We placed blood and tissue specimens in cryovials and transported them in liquid nitrogen to a biosafety level 4 facility for −70°C storage until testing.

We performed serologic, molecular, and virologic testing as described previously (*12*) and used real-time quantitative reverse transcription PCR (qRT-PCR) targeting the MARV L and VP40 genes to identify MARV-positive bats (*12*). When performing qRT-PCR with serum samples, we used pooled samples from 3–5 bats. We attempted virus isolation with samples that were positive for the MARV genome. We sequenced virus genomes using the TruSeq RNA Access Kit (Illumina Inc., San Diego, CA, USA) with enrichment probes designed against multiple MARV strains, including the 1975 Ozolin strain, and sequenced on an Illumina MiSeq (*14*). We performed sequence alignment using MAFFT version 7.222 (https://mafft.cbrc.jp/alignment/software/) and phylogenetic analysis using MrBayes version 3.2.6 (http://mrbayes.sourceforge.net/download.php).

Mating in the Egyptian rousette bat colony at Matlapitsi Cave occurred during June through mid-September. The first neonates were observed in the second half of October. In December and January, almost all female bats captured carried an infant or were pregnant. Neonates were observed occasionally in March and April, outside the birthing season, suggesting asynchronous births. In previous studies, the Egyptian rousette bat population in Matlapitsi Cave was estimated to fluctuate from 3,270 to 9,000 bats, with the lowest numbers occurring during the winter months (*15*).

**Figure 1:**
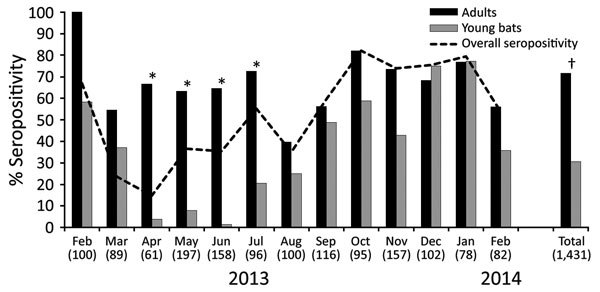
Marburg virus seropositivity in Egyptian rousette bats in Matlapitsi Cave, Limpopo Province, South Africa, 2013–2014. Numbers in parentheses indicate number of bats sampled per month. Bats <1 year of age (young bats) represent the new generation of bats born mostly during the October–January birthing peak. Statistically significant differences in seropositivity between adult and young bats are noted over a period of 4 months, April–July 2013. *Significant difference (p = 0.0001) between adult and young bat populations. †Significant difference (p = 0.002) between adult and young bat populations.

Of 1,431 bats tested, 759 (53.04%) were positive for antibodies against MARV; overall seropositivity ranged from 14.75% in April 2013 to 82.1% in October 2013. Seropositivity in adults (n = 784) was 71.56%, ranging from 39.6% in August 2013 to 100% in February 2013. Seropositivity in young bats (n = 647) was 30.6%, ranging from 1.3% in June 2013 to 77.3% in January 2014. Seropositivity was significantly higher in adult than young bats (p = 0.002), especially during April 2013–July 2013 (p = 0.0001) (Figure 1). In total, 45.3% of male bats (n = 592) and 58.5% of female bats (n = 839) were seropositive (p = 0.667). Seroconversion was detected in 12 (19.1%) of 63 recaptured bats. The bats that seroconverted were all juvenile bats on first capture (Table 1).

**Table 1:** Marburg virus seroconversion in 12 Egyptian rousette bats recaptured at Matlapitsi Cave, Limpopo Province, South Africa, April 2013–January 2014

Bat no.	First capture			Second capture			Third capture	
	ELISA, % positivity*	Capture date		ELISA, % positivity*	Capture date		ELISA, % positivity*	Capture date
243	8.48	2013 Mar 13		5.26	2013 May 13		67.15	2013 Aug 13
287	13.75	2013 Apr 13		45.67	2013 Nov 13			
310	7.11	2013 Apr 13		36.41	2013 Jul 13			
323	11.30	2013 Apr 13		53.94	2013 Feb 14			
525	6.22	2013 May 13		42.61	2013 Jun 13			
542	7.19	2013 May 13		67.97	2013 Aug 13		100.69	2013 Nov 13
615	4.91	2013 Jun 13		31.68	2013 Oct 13			
633	7.58	2013 Jun 13		53.93	2013 Jul 13			
653	4.53	2013 Jun 13		64.19	2013 Nov 13			
694	7.58	2013 Jun 13		4.24	2013 Sep 13		42.73	2013 Nov 13
742	5.72	2013 Jul 13		23.82	2013 Oct 13			
822	6.86	2013 Jun 13		41.97	2014 Jan 14			

*Percentage positivity of the internal positive control serum sample; cutoff value of assay is 16.78% positivity (*12*).

All serum pools tested by qRT-PCR were negative for both L and VP40 genes. Of the 159 liver-spleen tissue pools tested, 3 (1.89%) were positive for MARV RNA: specimen no. SPU191/13 from a juvenile female bat (no. 2,764) captured in July 2013 (cycle threshold [C_t_] L 29.84, C_t_ VP40 31.58); specimen no. SPU249/13 from a juvenile male bat (no. 3,003) captured in August 2013 (C_t_ L 34.86, C_t_ VP40 33.85); and specimen no. SPU282/13 from a juvenile male bat (no. 3,092) captured in September 2013 (C_t_ L 33.04, C_t_ VP40 34.05). Attempts to culture the virus from qRT-PCR–positive tissue pools were unsuccessful. A similar ecologic study conducted in Uganda obtained identical results from bat tissues with C_t_s >30 (*11*).

**Figure 2:**
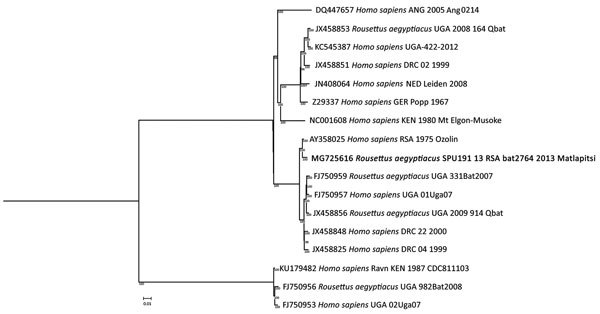
Phylogenetic tree of partial (97.5%) Marburg virus nucleic acid sequence detected in Egyptian rousette bats in Matlapitsi Cave, Limpopo Province, South Africa, 2013 (bold; GenBank accession no. MG725616) and complete nucleic acid sequences of representative Marburg virus strains from GenBank. Node values indicate posterior probability percentages obtained from 1,000,000 generations in MrBayes version 3.2.6 (http://mrbayes.sourceforge.net/download.php). Scale bar indicates nucleotide substitutions per site.

Genomic analysis was performed only with the specimen with the lowest qRT-PCR C_t_ (bat no. 2,764; SPU191/13). The MARV sequence detected (GenBank accession no. MG725616) was closely related to the 1975 Ozolin strain (99.3% nucleic acid homology, 0%–1.2% amino acid differences) (Table 2; Figure 2). Only 22 aa substitutions were identified between these 2 viruses, which were isolated 38 years apart.

**Table 2:** Base pair changes between reference MARV strains and MARV from Matlapitsi Cave, Limpopo Province, South Africa, 2013*

Sequence type, MARV gene	Strain, no. (%)			
	Ozolin 1975	Angola 2005	Musoke 1980	Uganda 2009
Nucleotide sequence				
Nucleocapsid	9 (0.43)	120 (5.8)	112 (5.4)	123 (5.9)
Viral protein 35	4 (0.43)	56 (5.7)	37 (3.7)	58 (5.9)
Viral protein 40	5 (0.57)	54 (5.9)	50 (5.5)	52 (5.7)
Glycoprotein	17 (0.85)	198 (9.7)	185 (9.0)	190 (9.3)
Viral protein 30	4 (0.48)	55 (6.5)	49 (5.8)	55 (6.5)
Viral protein 24	2 (0.26)	43 (5.7)	38 (5.0)	34 (4.4)
Polymerase	43 (0.6)	469 (6.7)	425 (6.1)	479 (6.8)
Amino acid sequence				
Nucleocapsid	0	11 (1.6)	12 (1.7)	11 (1.6)
Viral protein 35	0	4 (1.3)	1 (0.3)	4 (1.3)
Viral protein 40	1 (0.34)	4 (1.4)	3 (1.0)	3 (1.0)
Glycoprotein	8 (1.2)	60 (8.8)	61 (9.0)	63 (9.3)
Viral protein 30	1 (0.36)	8 (2.9)	13 (4.7)	10 (3.6)
Viral protein 24	0	2 (0.79)	2 (0.79)	1 (0.4)
Polymerase	12 (0.52)	83 (3.6)	87 (3.7)	87 (3.7)

*Matlapitsi Cave MARV sequences were from specimen no. SPU 191/13 from bat no. 2,764 (GenBank accession no. MG725616). MARV, Marburg virus.

## Conclusions

Our findings indicate endemic MARV circulation in bats in Matlapitsi Cave, located 111 km from Polokwane City, South Africa’s largest urban center north of Gauteng Province. Matlapitsi Cave, a 400-m hike from the main road running through the rural community of Fertilis, is accessible to humans. The cave was used in the past for religious practices and circumcision rituals, which have since been discontinued. In spite of their discontinuation, we found human shoe prints, litter, and signs of recent fire pit use at the cave entrance during our sampling trips. Informal discussions with persons of the local communities indicated that bats were not being hunted and their meat was not being consumed by local residents. However, uncontrolled migration of persons from neighboring countries where bat meat is consumed and increasing economic pressures, which could force local persons to hunt wildlife, might put the population at increased risk for MVD.

Observations made in this study confirm a distinctly seasonal Egyptian rousette bat reproductive period as previously reported (*15*). Gradual loss of passive immunity increases the number of susceptible bats, thus creating suitable conditions for MARV spread in the colony. Results of our study suggest that the single but relatively long birthing season complemented by asynchronous births and potential migration of bats might contribute to sustained annual MARV circulation in this area (*15*). These findings appear to be in contrast with those from the study in Uganda, which indicated that 2 yearly birthing seasons were required to maintain circulation of MARV in Egyptian rousette bats (*11*). The period of lowest seropositivity in young bats (April–July) might indicate a period of increased risk for exposure and shedding. The MARV sequence from the Matlapitsi Cave is phylogenetically most closely related to the Ozolin MARV strain, suggesting this variant has persisted in the southern part of Africa relatively unchanged since first discovered in 1975 (*3*). These findings contribute to our knowledge of MARV ecologic factors that could lead to a zoonotic spillover into humans and, thus, assist in the development of evidence-based policies for MVD risk reduction in South Africa.
